# Compressão Coronária na Hipertensão Pulmonar: Uma Ameaça Tratável Escondida à Vista de Todos

**DOI:** 10.36660/abc.20250352

**Published:** 2025-07-31

**Authors:** Allan K. N. Alencar

**Affiliations:** 1 Tulane University Department of Biomedical Engineering New Orleans EUA Department of Biomedical Engineering, Tulane University, New Orleans – EUA

**Keywords:** Hipertensão Pulmonar, Intervenção Coronária Percutânea, Angioplastia

A hipertensão pulmonar (HP) representa uma condição grave e progressiva associada à insuficiência ventricular direita, comprometimento da capacidade funcional e aumento da mortalidade.^1 , 2^ Embora a angina não seja um sintoma característico da HP, ela pode afetar até 30% dos pacientes e é frequentemente esquecida na prática clínica.^3^ O estudo publicado nesta edição dos Arquivos Brasileiros de Cardiologia^4^ aborda uma causa rara, porém crítica, de dor torácica na HP: compressão extrínseca do tronco da artéria coronária esquerda (TCE) por uma artéria pulmonar dilatada. Os autores apresentam uma série de casos observacionais de 12 pacientes submetidos à intervenção coronária percutânea (ICP) com implante de stent para compressão da TCE, relatando viabilidade e alívio sustentado dos sintomas.

Embora a aterosclerose do TCE seja bem reconhecida por seu impacto prognóstico, a compressão deste vaso devido à dilatação da artéria pulmonar na HP permanece subdiagnosticada. Os autores destacam que até 76% dos pacientes com HP avançada apresentam dilatação da artéria pulmonar, o que pode resultar em compressão extrínseca significativa do TCE, mesmo na ausência de aterosclerose coronariana.^4 , 5^ É importante ressaltar que a compressão do TCE tem sido associada à morte cardíaca súbita em coortes de HP, enfatizando a necessidade de alta suspeita clínica.^6 - 8^

O estudo atual reforça descobertas de relatórios anteriores, como a série italiana de Saia et al.,^9^ que demonstrou resultados positivos após a colocação de stent em 53 pacientes. Nesta série brasileira, todos os 12 pacientes relataram melhora dos sintomas, com sucesso angiográfico e sem complicações imediatas. Ao longo de um acompanhamento de 33 meses, ocorreram apenas quatro mortes, todas atribuídas à progressão da HP subjacente, sem relatos de eventos relacionados ao stent.

Esta série de casos é valiosa por vários motivos. Primeiro, ela fornece evidências do mundo real de um centro de HP de alto volume na América Latina, uma região sub-representada na pesquisa em HP coronária-vascular. Segundo, ela demonstra um algoritmo diagnóstico e terapêutico estruturado que envolve avaliação multidisciplinar, imagens avançadas com angiotomografia computadorizada de coronárias e tomada de decisão individualizada — um modelo que vale a pena replicar em centros especializados.

O estudo também se alinha à crescente conscientização sobre causas não ateroscleróticas de isquemia miocárdica em doenças sistêmicas. A fisiopatologia distinta da HP contribui para a subperfusão coronária devido ao aumento do estresse da parede do ventrículo direito e à alteração da reserva coronária.^9^ A adição de compressão mecânica pelo tronco pulmonar cria um mecanismo de "duplo impacto" que pode passar inadvertido, a menos que exames de imagem coronária sejam realizados de forma proativa em pacientes sintomáticos.

É importante destacar que os autores descrevem as nuances técnicas da seleção e implantação do stent. Na maioria dos casos, um único stent foi suficiente; no entanto, em 25% dos casos, um segundo stent foi necessário para otimizar a força radial e o ganho de lúmen. Embora a imagem intravascular tenha sido utilizada com parcimônia, sua aplicação seletiva ressalta as limitações do mundo real e a necessidade de abordagens flexíveis e pragmáticas em cenários com recursos limitados.

Uma das principais mensagens é que a compressão do TCE deve ser sistematicamente considerada em pacientes com HP e angina ou disfunção ventricular inexplicada. Embora as estratégias de revascularização para a doença aterosclerótica do TCE estejam bem estabelecidas,^10^ o papel da ICP na compressão extrínseca ainda está em evolução. O impacto prognóstico a longo prazo do implante de stent neste contexto não é totalmente conhecido, e o estudo atual se soma a um crescente corpo de literatura que sugere benefício sintomático, embora estudos randomizados sejam improváveis devido à raridade da condição.

Pesquisas futuras devem explorar a utilidade da triagem de rotina em pacientes com HP de alto risco, especialmente aqueles com diâmetros de artéria pulmonar > 40 mm ou proximidade anatômica entre a artéria pulmonar e o TCE em exames de imagem.^11^ Além disso, avaliar o impacto da ICP na função do ventrículo direito e na sobrevida a longo prazo pode gerar insights sobre o tratamento integrativo da HP.^12^

Em conclusão, os autores fornecem evidências convincentes de que a compressão do TCE na HP é clinicamente significativa e terapeuticamente viável. Sua experiência corrobora a incorporação da avaliação coronária aos algoritmos de tratamento da HP e posiciona a ICP como uma intervenção segura e eficaz em casos selecionados. Na era da cardiologia de precisão, reconhecer e tratar esses culpados anatômicos não só é viável, como também pode salvar vidas.
