# Supplementary material for: Compressão Coronária na Hipertensão Pulmonar: Uma Ameaça Tratável Escondida à Vista de Todos
Source: Arq Bras Cardiol. 2025 Jul 31;122(7):e20250352. [Article in Portuguese] doi: 10.36660/abc.20250352 (PMC12695067; doi:10.36660/abc.20250352)
Supplement: Translation

# Translation: Coronary Compression in Pulmonary Hypertension: A Treatable Threat Hidden in Plain Sight

**DOI:** 10.36660/abc.20250352i

**Authors:** Allan K. N. Alencar

**Affiliations:** 1 New Orleans USA Department of Biomedical Engineering, Tulane University, New Orleans – USA

**Keywords:** Pulmonary Hypertension, Percutaneous Coronary Intervention, Angioplasty

Pulmonary hypertension (PH) represents a severe, progressive condition associated with right ventricular failure, impaired functional capacity, and increased mortality.^1 , 2^Although angina is not a hallmark symptom of PH, it can affect up to 30% of patients and is frequently overlooked in clinical practice.^3^The study published in this issue of *Arquivos Brasileiros de Cardiologia* ^4^addresses a rare yet critical cause of chest pain in PH: extrinsic compression of the left main coronary artery (LMCA) by a dilated pulmonary artery. The authors present an observational case series of 12 patients undergoing percutaneous coronary intervention (PCI) with stenting for LMCA compression, reporting both feasibility and sustained symptom relief.

While LMCA atherosclerosis is well recognized for its prognostic impact, compression of this vessel due to pulmonary artery dilation in PH remains underdiagnosed. The authors highlight that up to 76% of patients with advanced PH exhibit pulmonary artery enlargement, which may result in significant extrinsic compression of the LMCA, even in the absence of coronary atherosclerosis.^4 , 5^Importantly, LMCA compression has been associated with sudden cardiac death in PH cohorts, emphasizing the need for high clinical suspicion.^6 - 8^

The current study reinforces findings from previous reports, such as the Italian series by Saia et al.^9^which demonstrated successful outcomes following stenting in 53 patients. In this Brazilian series, all 12 patients reported symptom improvement, with angiographic success and no immediate complications. Over a 33-month follow-up, only four deaths occurred, all attributed to the progression of underlying PH, with no stent-related events reported.

This case series is valuable for several reasons. First, it provides real-world evidence from a high-volume PH center in Latin America, a region underrepresented in coronary-vascular PH research. Second, it demonstrates a structured diagnostic and therapeutic algorithm involving multidisciplinary evaluation, advanced imaging with coronary computed tomography angiography, and individualized decision-making — a model worth replicating in specialized centers.

The study also aligns with growing awareness of non-atherosclerotic causes of myocardial ischemia in systemic diseases. The distinct pathophysiology of PH contributes to coronary underperfusion due to increased right ventricular wall stress and altered coronary reserve.^9^The addition of mechanical compression by the pulmonary trunk creates a ‘double-hit’ mechanism that may go unrecognized unless coronary imaging is proactively pursued in symptomatic patients.

Importantly, the authors describe technical nuances of stent selection and deployment. In most cases, a single stent sufficed; however, in 25% of cases, a second stent was required to optimize radial force and lumen gain. Although intravascular imaging was used sparingly, its selective application underscores the real-world constraints and the need for flexible, pragmatic approaches in resource-limited settings.

One of the key messages is that LMCA compression should be systematically considered in PH patients with angina or unexplained ventricular dysfunction. While revascularization strategies for atherosclerotic LMCA disease are well established,^10^the role of PCI in extrinsic compression is still evolving. The long-term prognostic impact of stenting in this context is not fully known, and the current study adds to a growing body of literature suggesting symptomatic benefit, though randomized studies are unlikely due to the rarity of the condition.

Future research should explore the utility of routine screening in high-risk PH patients, especially those with pulmonary artery diameters >40 mm or anatomic proximity between the pulmonary artery and LMCA on imaging.^11^Additionally, assessing the impact of PCI on right ventricular function and long-term survival could yield insights into the integrative management of PH.^12^

In conclusion, the authors provide compelling evidence that LMCA compression in PH is both clinically significant and therapeutically actionable. Their experience supports the incorporation of coronary evaluation into PH care algorithms and positions PCI as a safe and effective intervention in selected cases. In an era of precision cardiology, recognizing and treating such anatomical culprits is not only feasible — it may also prove to be life-saving.
